# Beyond surgical risk: Multidimensional predictors of postoperative arm morbidity after breast cancer treatment

**DOI:** 10.1016/j.breast.2026.104846

**Published:** 2026-06-20

**Authors:** Ifat Klein, Danit R. Shahar, Michael Friger, Irena Rosenberg, Sergio Susmallian, Daphna Barsuk, Merav A. Ben-David

**Affiliations:** a Department of Physical Therapy, Assuta Medical Centers, Tel Aviv, Israel; b Department of Epidemiology, Biostatistics and Community Health, School of Public Health, Faculty of Health Sciences, Ben-Gurion University of the Negev, Beersheva, Israel; c Department of Surgery, Assuta Medical Centers, Tel Aviv, Israel; d Department of Oncology, Faculty of Health Sciences, Ben-Gurion University of the Negev, Beersheva, Israel; e Department of Oncology, Assuta Medical Centers, Tel Aviv, Israel

## Abstract

**Background:**

Postoperative upper-limb morbidity following breast cancer treatment is common and clinically significant, encompassing pain, functional impairment, range-of-motion limitation, and lymphedema. While traditional risk models have focused primarily on surgical and treatment-related factors, less is known about the relative contribution of psychosocial and behavioral determinants. The Arm Morbidity following Breast Cancer Treatment (ARM-BCT) tool was developed as a clinically practical risk stratification instrument; however, its performance and underlying predictors require further evaluation in larger and more heterogeneous populations.

**Objectives:**

To identify multidimensional predictors of postoperative upper-limb morbidity following breast cancer treatment and evaluate the relative contribution of psychosocial, behavioral, clinical, and treatment-related factors across complementary analytical approaches.

**Methods:**

This pooled analysis included 1602 women assessed 0–36 months following breast cancer surgery across three prospective cohorts. Postoperative morbidity was defined as a composite outcome including pain, range-of-motion limitation, functional impairment, or lymphedema. Multivariable logistic regression and complementary machine-learning approaches were applied to evaluate the relative contribution of clinical, treatment-related, psychosocial, and lifestyle-related factors to postoperative morbidity. Model discrimination was assessed using the area under the receiver operating characteristic curve (AUC).

**Results:**

Postoperative morbidity was observed in 61% of participants. Across analytical approaches, psychosocial and behavioral factors demonstrated consistent associations with postoperative morbidity alongside several clinical and treatment-related variables. Insomnia (OR 2.91, 95% CI 2.19–3.87) and emotional distress (OR 1.18 per unit increase, 95% CI 1.12–1.25) were independently associated with increased morbidity, along with comorbidity (OR 1.80, 95% CI 1.37–2.36) and chemotherapy exposure (OR 2.01, 95% CI 1.39–2.89). Higher physical activity levels during survivorship follow-up were also associated with morbidity, although this relationship may partly reflect reverse causation. Several traditional surgical variables demonstrated relatively limited independent associations after multivariable adjustment compared with the multidimensional psychosocial, behavioral, and treatment-related framework. The ARM-BCT score demonstrated modest discrimination (AUC 0.68), whereas multivariable regression and complementary machine-learning approaches demonstrated improved discrimination (AUC 0.79–0.81).

**Conclusions:**

Postoperative arm morbidity following breast cancer treatment appears to reflect a multidimensional survivorship outcome influenced by psychosocial, behavioral, clinical, and treatment-related factors. While machine-learning approaches provided modest improvements in predictive performance, their primary contribution was in refining the interpretation of complex interactions between predictors. Integrating psychosocial and behavioral dimensions into postoperative risk assessment may support more personalized and proactive rehabilitation-oriented survivorship care strategies.

## Highlights

• Postoperative arm morbidity affected 61% of breast cancer survivors.
• Insomnia independently predicted morbidity (OR 2.91, 95% CI 2.19–3.87).
• Anxiety increased morbidity risk by 18% per score increase.
• Machine-learning models improved prediction (AUC 0.79–0.81 vs 0.68).
• Psychosocial and behavioral factors contributed to morbidity prediction.

## 1 Introduction

Upper-limb morbidity following breast cancer treatment is a common and clinically relevant survivorship complication encompassing pain, restricted shoulder mobility, functional impairment, and lymphedema [[1], [2], [3]]. Some impairments develop immediately after surgery, whereas others may evolve gradually over time during recovery and adjuvant treatment [[4], [5], [6]]. Although morbidity severity varies considerably, even relatively mild symptoms may affect participation in physical activity, return to routine, and overall functional recovery [7,8].

Traditional models of postoperative arm morbidity have focused primarily on surgical exposure and oncologic treatment factors, including axillary surgery, radiation therapy, and chemotherapy. However, recovery following breast cancer treatment is increasingly recognized as a multidimensional survivorship process influenced not only by clinical variables, but also by behavioral adaptation, symptom burden, emotional distress, sleep disturbance, and coping capacity [9,10]. Consequently, postoperative morbidity may reflect broader patient-related vulnerability rather than surgical exposure alone [11,12]. Improved risk assessment may therefore require more comprehensive frameworks integrating psychosocial and behavioral dimensions alongside traditional clinical factors [13].

To support early identification of women at increased risk for postoperative morbidity, the Arm Morbidity following Breast Cancer Treatment (ARM-BCT) tool was developed as a clinically applicable multidimensional screening instrument intended for use beginning in the immediate postoperative period [14]. The framework integrates surgical, treatment-related, behavioral, and psychosocial factors into a cumulative risk score designed to support more personalized rehabilitation recommendations and survivorship follow-up.

Because postoperative recovery trajectories are heterogeneous and may involve complex interactions between clinical and patient-related factors, complementary data-driven approaches may provide additional insight into multidimensional risk patterns [15]. In the current study, machine-learning methods were applied primarily as exploratory and complementary analytical tools to evaluate the relative contribution of predictors included within the ARM-BCT framework and to examine whether consistent multidimensional patterns emerged across analytical approaches [16,17].

Therefore, the aims of the current study were to evaluate multidimensional predictors of postoperative upper-limb morbidity following breast cancer treatment within a large pooled cohort, to assess the relative contribution of psychosocial, behavioral, clinical, and treatment-related factors, and to examine whether complementary analytical approaches demonstrate consistent patterns of risk stratification across survivorship recovery trajectories.

## 2 Methods

### 2.1 Study design and setting

This study is a pooled analysis of three prospective observational cohorts conducted between 2023 and 2024 at tertiary medical centers in Israel. These cohorts included: (1) a development cohort focused on derivation of the ARM-BCT screening tool, (2) a validation cohort evaluating the discriminative performance of the screening tool against objective functional assessments, and (3) an implementation cohort evaluating rehabilitation utilization following risk-based recommendations.

The combined analytic sample included 1602 women assessed between 0 and 36 months following breast cancer surgery. Across cohorts, harmonized inclusion and exclusion criteria, standardized morbidity definitions, and comparable data collection procedures for clinical, treatment-related, psychosocial, behavioral, and outcome variables were applied, enabling integration into a unified analytical dataset.

### 2.2 Ethical approval

The study was approved by the institutional review board of Assuta Medical Centers (ASMC-0018-23 and ASMC-0019-22), and all participants provided informed consent for participation and use of anonymized clinical and questionnaire data for research purposes, including pooled analyses within the ARM-BCT research program. The study was prospectively registered at ClinicalTrials.gov (NCT05950685).

### 2.3 Participants

Eligible participants included women aged 18–80 years who underwent oncologic breast surgery, including lumpectomy or mastectomy, with or without axillary intervention (sentinel lymph node biopsy or axillary lymph node dissection).

Exclusion criteria were metastatic disease at the time of surgery, benign breast pathology, pre-existing lymphedema, additional ipsilateral surgery or injury affecting arm function, and severe cognitive impairment. Participants were included in the analytic cohort if they completed at least one postoperative morbidity assessment.

### 2.4 Outcome definition

The primary outcome was postoperative upper-limb morbidity, defined as a composite binary measure reflecting the presence of at least one of four clinically relevant domains: pain, range of motion limitation, functional impairment, or lymphedema.

Pain was assessed using the Numeric Pain Rating Scale (0–10) and was considered clinically significant if the reported score was greater than 1 during the past week [18].

Range-of-motion limitation was defined as self-reported inability to achieve full overhead elevation of the affected upper limb [19].

Functional impairment was assessed using the Quick Disabilities of the Arm, Shoulder and Hand (QuickDASH) questionnaire (score range: 0–100) [20,21]. A threshold of ≥20 was used to identify clinically meaningful upper-limb functional limitation during survivorship follow-up.

Lymphedema was defined as either clinician-diagnosed upper-limb lymphedema documented in the medical record or self-reported persistent swelling involving the arm, chest, or axillary region [22,23].

Participants were classified as having postoperative arm morbidity if at least one of these domains was present. The composite outcome was selected to reflect the overall multidimensional burden of postoperative upper-limb morbidity relevant to survivorship recovery and rehabilitation utilization rather than to distinguish severity levels of individual impairments.

### 2.5 Predictor variables

Predictor variables were selected to reflect the multidimensional nature of postoperative recovery and included demographic, clinical, treatment-related, behavioral, and psychosocial domains.

Demographic variables included age (years) and body mass index (BMI, kg/m^2^).

Clinical and surgical variables included type of breast surgery (lumpectomy or mastectomy), extent of axillary surgery (none, sentinel lymph node biopsy, or axillary dissection), breast reconstruction (yes/no), and tumor stage (in situ, localized invasive, or advanced disease).

Treatment-related variables included receipt of chemotherapy, radiation therapy, hormonal therapy, and biological therapy (all coded as binary variables), as well as BRCA mutation status (yes/no).

Postoperative variables included the presence of complications (yes/no) and severe postoperative pain during hospitalization, defined as a Numeric Pain Rating Scale score ≥6. Receipt of physiotherapy during hospitalization was recorded as a binary variable (yes/no).

Behavioral and psychosocial variables included physical activity, psychological distress, depressive symptoms, insomnia, and perceived family support.

Physical activity was assessed during survivorship follow-up using a questionnaire based on the Godin–Shephard Leisure-Time Exercise framework and was analyzed as a behavioral correlate of postoperative recovery status rather than a strictly preoperative predictive factor [24]. To enhance clinical usability, responses were categorized into three levels (high, moderate, low) and coded as an ordinal variable (−1 = high activity, 0 = moderate activity, 1 = low activity), with higher values indicating lower levels of physical activity.

Emotional distress was assessed using a 0–10 numeric rating scale reflecting anxiety experienced during the preceding week, with higher scores indicating greater emotional distress.

#### 2.5.1 ARM-BCT assessment

The Arm Morbidity following Breast Cancer Treatment (ARM-BCT) score was calculated for all participants. This clinically applicable 17-item multidimensional screening framework integrates clinical, treatment-related, behavioral, and psychosocial factors into a cumulative risk score intended to support early identification of women at increased risk for postoperative upper-limb morbidity and to facilitate personalized rehabilitation follow-up.

In the current analysis, the ARM-BCT score was evaluated as a clinically derived multidimensional risk framework in separate models. To avoid multicollinearity and conceptual overlap, the composite score was not included simultaneously with its individual component variables in multivariable analyses.

### 2.6 Statistical analysis

Descriptive statistics were used to summarize cohort characteristics, with continuous variables presented as medians and interquartile ranges (IQRs) and categorical variables as frequencies and percentages. Group comparisons were performed using the Mann–Whitney *U* test for continuous variables and the chi-square test for categorical variables.

Multicollinearity among predictors was assessed using variance inflation factors (VIFs). Multivariable logistic regression analyses were performed to evaluate independent associations between clinical, treatment-related, behavioral, and psychosocial factors and the composite postoperative arm morbidity outcome while accounting for clinically relevant covariates. Results are presented as odds ratios (ORs) with 95% confidence intervals (CIs).

All statistical analyses were performed using SPSS version 30 (IBM Corp., Armonk, NY, USA) and R version 4.4.2 (R Foundation for Statistical Computing, Vienna, Austria). A two-sided p-value <0.05 was considered statistically significant.

#### 2.6.1 Machine learning analysis

Complementary machine-learning analyses were performed to further evaluate multidimensional patterns of postoperative morbidity beyond conventional regression modeling. These analyses were applied primarily to examine the relative contribution and interaction of clinical, treatment-related, behavioral, and psychosocial factors across different analytical frameworks rather than to replace clinically interpretable models.

The analytical framework included multivariable logistic regression, random forest, and Extreme Gradient Boosting (XGBoost) models. The dataset was randomly divided into training (70%) and testing (30%) subsets using stratified sampling based on the outcome distribution.

Model discrimination was evaluated using the area under the receiver operating characteristic curve (AUC), along with sensitivity, specificity, positive predictive value (PPV), negative predictive value (NPV), accuracy, and F1 score. Feature importance analyses and SHAP (Shapley Additive Explanations) values were used to support interpretation of predictor contribution across models.

### 2.7 Sample size considerations

Given that this study represents a secondary analysis of pooled prospective cohorts, no a priori sample size calculation was performed for the current analysis. Instead, the available sample size of 1602 participants, with an observed morbidity prevalence of approximately 60%, provides a large number of outcome events sufficient for both multivariable regression and machine-learning approaches.

## 3 Results

### 3.1 Participant characteristics and morbidity status

A total of 1602 women were included in the pooled analysis (Supplementary Fig. S1). The median age of the cohort was 58 years (IQR 50–67), and the median body mass index (BMI) was 25.8 kg/m^2^ (IQR 22.7–29.3).

During follow-up, 978 women (61.0%) developed postoperative upper-limb morbidity. Women with morbidity differed from those without morbidity across several clinical, treatment-related, behavioral, and psychosocial characteristics. Morbidity was more common among women with comorbid conditions, postoperative complications, advanced disease stage, and exposure to adjuvant treatments including chemotherapy, radiation therapy, hormonal therapy, and biological therapy. Insomnia was substantially more prevalent among women with morbidity.

Higher levels of physical activity during survivorship follow-up were more frequently observed among women with morbidity, whereas low physical activity levels were more common among women without morbidity. Age and BMI did not differ significantly between groups. Detailed participant characteristics are presented in Table 1.

**Table 1:** Participant characteristics by postoperative arm morbidity status.

Variable	No morbidity	Any morbidity	p value
Age, years (median, IQR)	59.0 (50.0–68.0)	58.0 (50.0–67.0)	0.472

BMI, kg/m² (median, IQR)	25.7 (22.5–29.4)	25.8 (22.8–29.3)	0.880

Stage			<0.001
In situ	343 (48.4%)	170 (19.9%)	
Localized disease	344 (48.5%)	622 (72.7%)	
Advanced disease	22 (3.1%)	63 (7.4%)	

Type of surgery			<0.001
Lumpectomy	389 (72.8%)	663 (78.6%)	
Mastectomy	145 (27.2%)	180 (21.4%)	

Extent of axillary surgery			<0.001
No nodes removed	176 (24.8%)	307 (35.9%)	
1–4 nodes removed	477 (67.3%)	428 (50.1%)	
5–12 nodes removed	56 (7.9%)	120 (14.0%)	

Breast reconstruction			0.002
No reconstruction	636 (89.7%)	721 (84.3%)	
Reconstruction	73 (10.3%)	134 (15.7%)	

Comorbidity			<0.001
No comorbidity	520 (73.3%)	439 (51.3%)	
Comorbidity present	189 (26.7%)	416 (48.7%)	

Chemotherapy			<0.001
No chemotherapy	631 (89.0%)	633 (74.0%)	
Chemotherapy	78 (11.0%)	222 (26.0%)	

Radiation therapy			<0.001
No radiation	531 (74.9%)	511 (59.8%)	
Radiation therapy	178 (25.1%)	344 (40.2%)	

Hormonal therapy			<0.001
No hormonal therapy	520 (73.3%)	460 (53.8%)	
Hormonal therapy	189 (26.7%)	395 (46.2%)	

Biological therapy			0.002
No biological therapy	631 (89.0%)	713 (83.4%)	
Biological therapy	78 (11.0%)	142 (16.6%)	

Postoperative complications			<0.001
No complications	657 (92.7%)	681 (79.6%)	
Complications present	52 (7.3%)	174 (20.4%)	

Physical activity level			<0.001
High	146 (20.6%)	323 (37.8%)	
Moderate	104 (14.7%)	208 (24.3%)	
Low	459 (64.7%)	324 (37.9%)	

Insomnia			<0.001
No insomnia	604 (85.2%)	420 (49.1%)	
Insomnia present	105 (14.8%)	435 (50.9%)	

**Abbreviations:** BMI, body mass index; IQR, interquartile range.

**Footnote:** Values are presented as n (%) for categorical variables and median (interquartile range) for continuous variables. Between-group comparisons were performed using the chi-square test for categorical variables and the Mann–Whitney *U* test for continuous variables. Postoperative arm morbidity was defined as the presence of at least one of the following: pain, range of motion limitation, functional impairment, or lymphedema. Comorbidity was defined as the presence of at least one neurological or orthopedic condition affecting function, prior ipsilateral shoulder surgery or injury, fibromyalgia, or chronic pain syndrome. Physical activity was assessed during survivorship follow-up.

### 3.2 Univariate predictors of postoperative arm morbidity domains

Univariate analyses were performed to examine associations between individual predictors and each domain of postoperative arm morbidity, including pain, range-of-motion limitation, functional impairment, and lymphedema.

Several predictors demonstrated consistent associations across morbidity domains. Postoperative complications and adjuvant treatments, including chemotherapy, radiation therapy, and hormonal therapy, were associated with increased odds across outcomes. Psychosocial and behavioral variables, particularly insomnia and psychological distress, demonstrated some of the strongest associations with pain, range-of-motion limitation, functional impairment, and lymphedema. In contrast, demographic variables such as age and BMI demonstrated relatively limited associations across morbidity domains. Detailed univariate associations are presented in Table 2.

**Table 2:** Associations between clinical predictors and postoperative arm morbidity domains.

Variable	Pain OR (95% CI)	ROM limitation OR (95% CI)	Functional impairment OR (95% CI)	Lymphedema OR (95% CI)
Age (years)	1.00 (0.99–1.01)	1.00 (0.99–1.01)	1.00 (0.99–1.01)	1.01 (0.99–1.03)
BMI (kg/m²)	0.99 (0.97–1.01)	1.02 (0.99–1.04)	1.00 (0.98–1.03)	**1.04 (1.00**–**1.08)**
Comorbidity (yes vs no)	**1.93 (1.54**–**2.43)**	1.23 (0.98–1.56)	0.92 (0.73–1.16)	0.88 (0.57–1.36)
Breast reconstruction (yes vs no)	1.14 (0.84–1.56)	**1.76 (1.29**–**2.39)**	**1.48 (1.09**–**2.01)**	1.25 (0.71–2.19)
Chemotherapy (yes vs no)	**1.88 (1.41**–**2.51)**	**2.55 (1.95**–**3.32)**	**2.40 (1.84**–**3.12)**	**2.47 (1.58**–**3.85)**
Radiation therapy (yes vs no)	**1.50 (1.19**–**1.89)**	**2.31 (1.82**–**2.93)**	**2.19 (1.74**–**2.77)**	**2.42 (1.57**–**3.73)**
Hormonal therapy (yes vs no)	**1.73 (1.38**–**2.18)**	**1.89 (1.49**–**2.39)**	**2.11 (1.68**–**2.66)**	**2.06 (1.33**–**3.18)**
Biological therapy (yes vs no)	1.25 (0.92–1.71)	**1.52 (1.12**–**2.05)**	**1.54 (1.15**–**2.08)**	**1.55 (0.92**–**2.61)**
Postoperative complications (yes vs no)	**2.16 (1.54**–**3.02)**	**2.39 (1.78**–**3.21)**	**3.29 (2.45**–**4.42)**	**3.03 (1.91**–**4.81)**
Insomnia (yes vs no)	**4.50 (3.46**–**5.86)**	**3.96 (3.10**–**5.05)**	**4.22 (3.32**–**5.37)**	**2.18 (1.42**–**3.36)**
Psychological distress (continuous)	**2.43 (1.87**–**3.15)**	**3.18 (2.49**–**4.07)**	**4.17 (3.26**–**5.33)**	**2.65 (1.72**–**4.07)**
Lack of family support (yes vs no)	1.41 (0.94–2.13)	**1.84 (1.25**–**2.69)**	**2.25 (1.54**–**3.28)**	**1.86 (1.00**–**3.46)**
Physiotherapy during hospitalization (yes vs no)	**1.29 (1.03**–**1.62)**	**1.28 (1.01**–**1.62)**	**1.27 (1.01**–**1.60)**	**1.71 (1.11**–**2.63)**
Stage	**p < 0.001**	**p < 0.001**	**p < 0.001**	**p = 0.008**
Type of surgery	**p < 0.001**	**p < 0.001**	**p < 0.001**	**p = 0.003**
Axillary surgery	**p < 0.001**	**p < 0.001**	**p < 0.001**	**p < 0.001**
Physical activity level	**p < 0.001**	**p < 0.001**	**p < 0.001**	p = 0.087

**Abbreviations:** BMI, body mass index; OR, odds ratio; CI, confidence interval; ROM, range of motion; QuickDASH, Quick Disabilities of the Arm, Shoulder and Hand questionnaire.

Values are presented as odds ratios (ORs) with 95% confidence intervals (CIs) derived from univariate logistic regression analyses for binary and continuous predictors. Models were analyzed separately for each morbidity domain and were not adjusted for potential confounders.

For variables with more than two categories (stage, type of surgery, axillary surgery, and physical activity level), overall associations are presented as p-values from chi-square analyses; category-specific ORs were not estimated. Functional impairment was defined as QuickDASH ≥20.

### 3.3 Multivariable predictors of postoperative arm morbidity

Multivariable logistic regression analysis was performed to identify independent predictors of postoperative arm morbidity (Table 3). No evidence of problematic multicollinearity was identified among predictors (all variance inflation factors <2). Several variables remained independently associated with increased morbidity risk after adjustment, including comorbidity, disease stage, chemotherapy exposure, postoperative complications, insomnia, and psychological distress. Detailed multivariable associations are presented in Table 3.

**Table 3:** Multivariable logistic regression analysis of predictors of postoperative arm morbidity among women undergoing breast cancer surgery.

**Variable**	**OR**	**95% CI**	**p-value**
Comorbidity (present vs none)	1.80	1.37–2.36	**<0.001**
Stage (per one-level increase)	1.99	1.56–2.55	**<0.001**
Stage (advanced vs no disease)	1.57	0.85–2.92	0.151
Mastectomy vs lumpectomy	0.70	0.48–1.01	0.056
Axillary surgery (1–4 nodes vs none)	0.77	0.57–1.03	0.08
Axillary surgery (≥5 nodes vs none)	0.85	0.54–1.31	0.454
Breast reconstruction (yes vs no)	1.41	0.92–2.18	0.117
Severe pain during hospitalization (yes vs no)	0.85	0.57–1.24	0.394
Chemotherapy (yes vs no)	2.01	1.39–2.89	**<0.001**
Radiation therapy (yes vs no)	0.93	0.68–1.28	0.662
Hormonal therapy (yes vs no)	1.31	0.86–1.55	0.069
Biological therapy (yes vs no)	0.78	0.54–1.13	0.195
Postoperative complications (yes vs no)	2.06	1.41–3.02	**<0.001**
Physical activity level (per one-level increase toward lower activity)	0.69	0.59–0.79	**<0.001**
Insomnia (yes vs no)	2.91	2.19–3.87	**<0.001**
Lack of family support (yes vs no)	1.10	0.69–1.77	0.682
Physiotherapy during hospitalization (yes vs no)	1.04	0.80–1.34	0.786
Age (years)	1.00	0.99–1.01	0.857
BMI (kg/m²)	1.00	0.98–1.03	0.936
Emotional distress (per unit increase)	1.18	1.12–1.25	**<0.001**

**Footnote:** Multivariable logistic regression analysis was performed to identify independent predictors of postoperative arm morbidity. Odds ratios (ORs) and 95% confidence intervals (CIs) are presented.

The dependent variable was postoperative arm morbidity, defined as the presence of at least one of the following: pain, range-of-motion limitation, functional impairment, or lymphedema. Emotional distress was assessed using a self-reported 0–10 numeric rating scale reflecting psychological distress experienced during the preceding week. Physical activity was modeled as an ordinal variable (−1 = high, 0 = moderate, 1 = low), such that higher values reflect lower activity levels. A two-sided p-value <0.05 was considered statistically significant.

Psychosocial and behavioral variables demonstrated some of the strongest independent associations with postoperative morbidity. Insomnia was associated with nearly threefold higher odds of morbidity, while higher levels of psychological distress were also independently associated with increased risk. Higher levels of physical activity during survivorship follow-up were associated with greater reported morbidity. This association should be interpreted cautiously and may reflect increased symptom awareness, higher functional demands, or reverse causation during survivorship recovery.

Most surgical variables, including type of surgery, extent of axillary surgery, and breast reconstruction, were not independently associated with postoperative morbidity after adjustment. Similarly, age, BMI, and several treatment-related variables, including radiation therapy and biological therapy, were not significantly associated with the outcome. Overall, psychosocial and behavioral variables demonstrated stronger independent associations with postoperative morbidity than most traditional surgical variables.

### 3.4 Model performance

The ARM-BCT score demonstrated modest discriminative performance for postoperative arm morbidity (AUC = 0.68), with relatively low sensitivity (0.55). In contrast, multivariable regression and machine-learning models demonstrated improved discrimination, with AUC values ranging from 0.79 to 0.81. Logistic regression achieved the highest sensitivity (0.89), whereas random forest demonstrated the highest overall AUC (0.81) and a more balanced sensitivity–specificity profile. XGBoost demonstrated comparable performance (AUC = 0.79).

Differences in predictive performance between logistic regression and machine-learning approaches were modest, suggesting that clinically interpretable multidimensional models captured much of the relevant prognostic information. Sensitivity analyses excluding hormonal therapy yielded similar model performance across analytical approaches, indicating that overall discrimination was not dependent on this variable. Internal train/test validation demonstrated similar model discrimination across analytical frameworks. Detailed performance metrics are presented in Table 4.

**Table 4:** Predictive performance of ARM-BCT and machine-learning models for postoperative arm morbidity.

Model	AUC	Cutoff	Sensitivity	Specificity	PPV	NPV	Accuracy	F1 score
ARM-BCT	0.68	7.0	0.54	0.70	0.69	0.56	0.62	0.61
Logistic regression	0.80	0.4	0.89	0.57	0.71	0.81	0.74	0.79
Random forest	0.81	0.5	0.80	0.71	0.77	0.75	0.76	0.78
XGBoost	0.79	0.5	0.77	0.70	0.76	0.72	0.74	0.76

**Abbreviations:** AUC, area under the receiver operating characteristic curve; PPV, positive predictive value; NPV, negative predictive value. Optimal cutoffs were determined using Youden's index.

Machine-learning models were evaluated using internal validation procedures based on train/test partitioning.

**Fig. 1:**
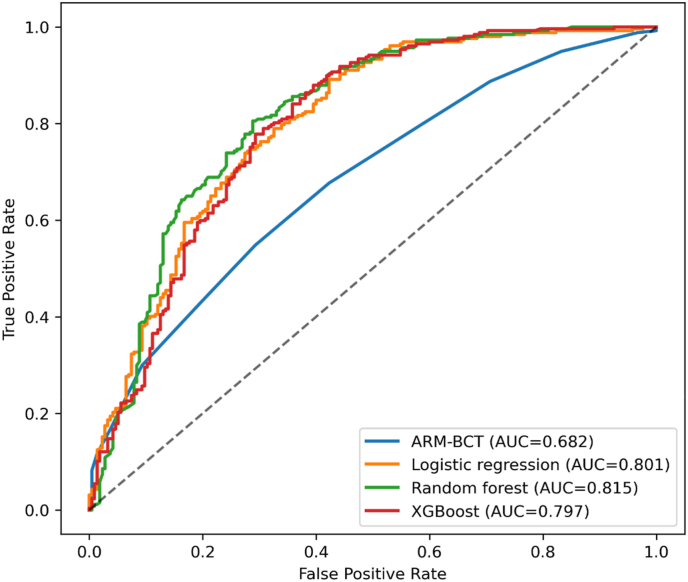
Receiver operating characteristic (ROC) curves comparing predictive models for postoperative arm morbidity ROC curves comparing the discriminative performance of the ARM-BCT score, multivariable logistic regression, random forest, and XGBoost models for predicting postoperative arm morbidity. Higher area under the curve (AUC) values indicates better overall model discrimination.

ROC curve analyses demonstrated similar discriminative performance across multivariable regression and machine-learning approaches, with all models outperforming the ARM-BCT score alone (Fig. 1).

### 3.5 Key predictors of postoperative arm morbidity

**Fig. 2:**
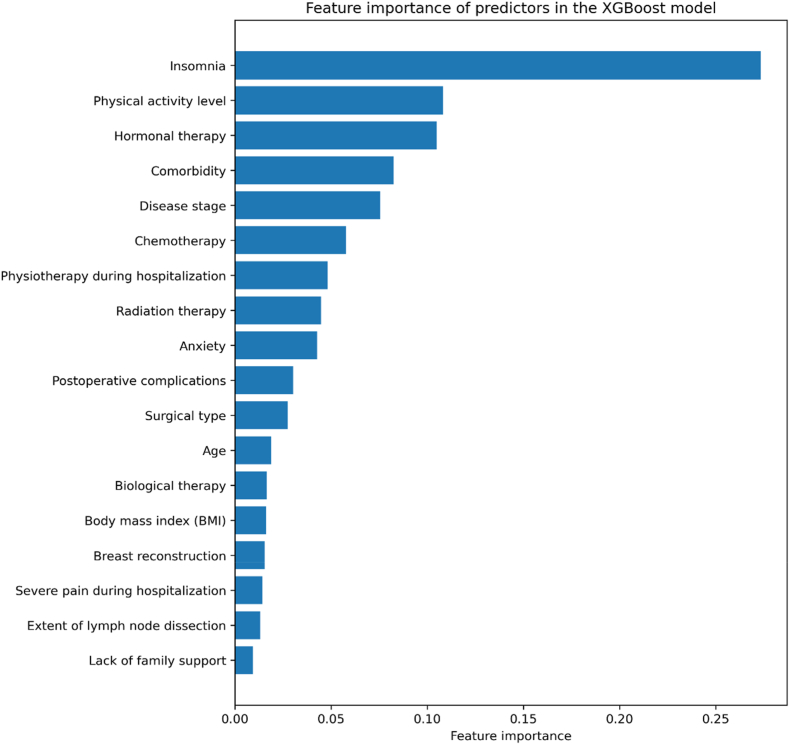
Relative feature importance of predictors in the XGBoost model for postoperative arm morbidity.

The relative importance of predictors in the XGBoost model is presented in Fig. 2. Insomnia emerged as the dominant predictor, demonstrating substantially greater importance than all other variables. Additional influential predictors included physical activity level, hormonal therapy, disease stage, comorbidity, and emotional distress.

Psychosocial and behavioral factors contributed prominently to model performance, whereas traditional surgical variables, including type of surgery and extent of axillary surgery, demonstrated comparatively lower importance. The full feature importance values are presented in Supplementary Table S1.

**Fig. 3:**
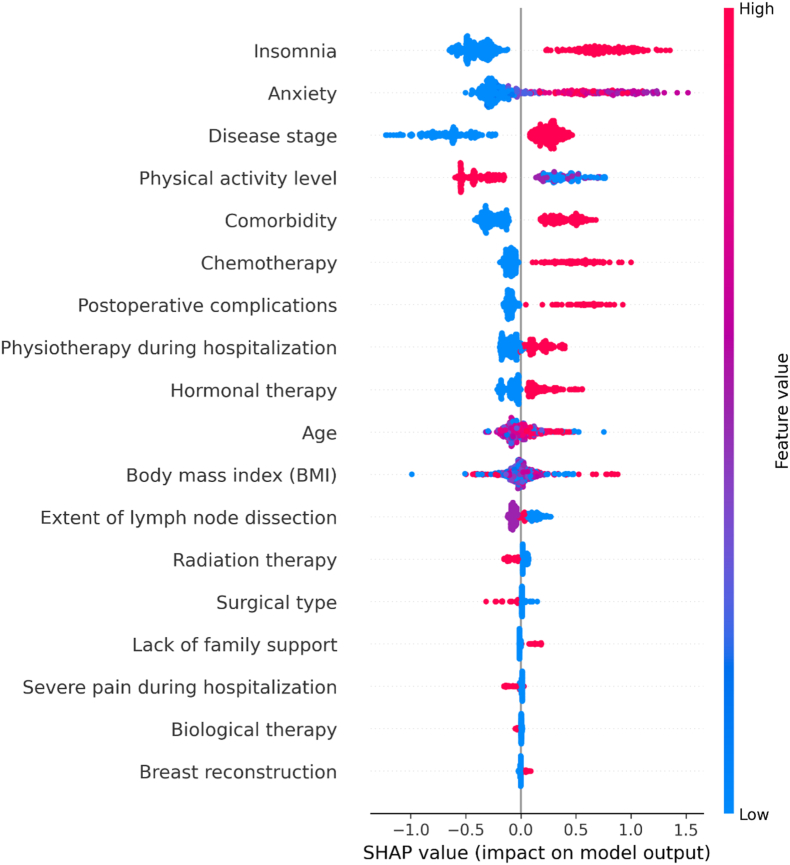
SHAP summary plot of predictors of postoperative arm morbidity SHAP (Shapley Additive Explanations) summary plot demonstrating the direction and magnitude of predictor contributions within the XGBoost model. Positive SHAP values indicate increased predicted morbidity risk, whereas negative values indicate lower predicted risk.

The contribution and direction of individual predictors within the XGBoost model are illustrated using SHAP (Shapley Additive Explanations) values in Fig. 3. Consistent with the feature importance analysis, insomnia demonstrated the strongest contribution to predicted postoperative morbidity risk. Emotional distress, physical activity level, disease stage, comorbidity, and chemotherapy exposure also contributed substantially to model predictions. In contrast, traditional surgical variables demonstrated relatively limited contributions across predictions.

The figure presents the relative contribution of each predictor to the discriminative performance of the XGBoost model. Higher values indicate greater influence on model predictions.

Fig. 3 illustrates the contribution and direction of individual predictors within the XGBoost model using SHAP (Shapley Additive Explanations) values. Consistent with the feature importance analysis, insomnia demonstrated the strongest contribution to predicted postoperative morbidity risk, followed by emotional distress, physical activity, disease stage, comorbidity, and chemotherapy exposure. Traditional surgical variables demonstrated comparatively lower contributions across predictions.

## 4 Discussion

The current study demonstrates that postoperative upper-limb morbidity following breast cancer treatment reflects a multidimensional survivorship outcome influenced by psychosocial, behavioral, clinical, and treatment-related factors. Traditionally, postoperative morbidity has been conceptualized primarily as a consequence of surgical exposure and treatment intensity, particularly axillary surgery, radiation therapy, and chemotherapy. Across multivariable and machine-learning approaches, psychosocial and behavioral variables demonstrated consistent and clinically meaningful associations with postoperative morbidity. Several treatment-related and clinical predictors, particularly chemotherapy exposure, comorbidity, and disease stage, also remained independently associated with morbidity outcomes. Postoperative arm morbidity remained highly prevalent throughout survivorship, supporting the view that rehabilitation-related impairments following breast cancer treatment represent persistent survivorship challenges rather than transient surgical complications alone [25]. Recovery after breast cancer treatment therefore appears to reflect a multidimensional process influenced not only by treatment-related factors [[26], [27], [28]], but also by symptom perception, psychological burden, coping capacity, and behavioral adaptation during survivorship [27,28].

Among all evaluated predictors, insomnia emerged as the most influential factor across both regression and machine-learning analyses. Sleep disturbance may reflect overlapping dimensions of recovery, including psychological distress, fatigue, persistent symptoms, impaired coping, and reduced resilience during survivorship. Emotional distress also demonstrated an independent association with morbidity outcomes. Although psychosocial variables are increasingly recognized within survivorship care, many traditional postoperative risk models continue to emphasize predominantly surgical and oncologic factors. The current findings suggest that psychosocial dimensions may warrant greater clinical attention during postoperative follow-up and rehabilitation assessment.

The association observed between physical activity and postoperative morbidity should be interpreted cautiously. Because physical activity was assessed during the postoperative period rather than before surgery, the observed relationship may partly reflect reverse causation. Patients experiencing morbidity may reduce or modify activity levels during recovery, while women with higher functional demands may also be more likely to perceive and report subtle limitations. Physical activity in this context may therefore represent a marker of functional adaptation and symptom burden rather than a direct causal predictor of morbidity [29].

In contrast to expectations based on previous literature, several surgical variables demonstrated relatively limited independent associations after multivariable adjustment within the multidimensional survivorship framework. Type of surgery, extent of axillary intervention, and breast reconstruction were not independently associated with postoperative morbidity within multivariable models [[30], [31], [32]]. These findings do not suggest that surgical factors are clinically unimportant, but rather that postoperative morbidity in contemporary breast cancer survivorship appears to reflect a broader recovery process extending beyond surgical exposure alone. Improvements in contemporary surgical techniques and perioperative management may also have reduced some of the long-term morbidity historically attributed to surgical treatment [27,33].

Machine-learning approaches demonstrated modestly improved predictive performance compared with the ARM-BCT score alone; however, performance remained broadly comparable to conventional multivariable logistic regression. These findings suggest that improved prediction may derive primarily from integrating multidimensional psychosocial, behavioral, and clinical information rather than from algorithmic complexity itself [16,34]. In the current study, machine-learning methods were used primarily as complementary analytical tools to characterize complex recovery patterns and explore interactions between predictors, rather than to replace clinically interpretable models [35].

Importantly, the consistent identification of psychosocial and behavioral variables across analytical approaches may have meaningful clinical implications. Factors such as sleep disturbance, emotional burden, coping difficulties, and changes in activity patterns are not routinely emphasized during standard postoperative follow-up, despite their potential contribution to long-term morbidity risk [11,36]. These dimensions may represent potentially modifiable targets for early supportive and rehabilitation-oriented interventions during breast cancer survivorship [37,38].

The ARM-BCT score demonstrated lower discriminative performance in the current pooled cohort compared with earlier validation analyses. This difference likely reflects the broader and more heterogeneous population included in the present analysis, which incorporated women across multiple postoperative stages and varying levels of symptom severity. Nevertheless, the ARM-BCT remains a clinically practical and interpretable screening tool that may support early identification of patients who could benefit from closer rehabilitation follow-up. While more complex machine-learning models demonstrated modest improvements in predictive performance, clinically practical tools remain essential for implementation in routine survivorship care.

Overall, the findings of the current study support a broader conceptualization of postoperative arm morbidity following breast cancer treatment as a multidimensional survivorship outcome shaped by interacting clinical, psychological, and behavioral factors [27,29]. Early identification of patients at increased risk may therefore require assessment frameworks extending beyond traditional surgical risk factors alone. Integrating psychosocial and behavioral dimensions into postoperative risk assessment may support more personalized and proactive survivorship care approaches aimed at improving long-term functional recovery after breast cancer treatment [39].

### 4.1 Limitations and strengths

Several limitations should be acknowledged. First, morbidity outcomes were assessed primarily using self-reported measures, which may be influenced by symptom perception, reporting behavior, and individual coping characteristics. Second, the composite morbidity outcome did not distinguish between different severity levels of postoperative impairment and may therefore have combined relatively mild symptoms with more clinically substantial functional limitations. Third, because the pooled cohort included women across different postoperative stages, temporal variability in recovery trajectories may have contributed to heterogeneity in morbidity patterns and predictor associations.

In addition, although the study incorporated prospective cohorts, several variables were evaluated cross-sectionally during survivorship follow-up, limiting causal interpretation, particularly for physical activity patterns where reverse causation is possible. In addition, the pooled analysis combined data from multiple cohorts with partially different study designs and postoperative assessment timeframes, which may have introduced additional heterogeneity despite harmonization efforts across datasets. Finally, external validation in independent populations is required before broader implementation of the machine-learning models.

Despite these limitations, the study has several important strengths. The analysis was based on a large pooled cohort with harmonized prospective data collection and standardized outcome definitions across cohorts. The multidimensional assessment framework enabled simultaneous evaluation of clinical, treatment-related, psychosocial, and lifestyle-related factors within a unified survivorship model. Furthermore, the integration of conventional regression approaches with machine-learning analyses allowed both clinically interpretable risk estimation and exploration of complex interactions between predictors, providing a broader understanding of postoperative recovery following breast cancer treatment.

## 5 Clinical implications

The findings of the current study suggest that postoperative morbidity risk assessment following breast cancer treatment may benefit from broader multidimensional evaluation extending beyond traditional surgical risk factors alone. Systematic assessment of sleep disturbance, psychological distress, and changes in activity patterns and functional adaptation may help identify patients at increased risk for persistent morbidity during survivorship. Incorporating these dimensions into postoperative follow-up and rehabilitation planning may support earlier and more personalized supportive interventions aimed at improving long-term functional recovery.

## 6 Conclusions

Postoperative arm morbidity following breast cancer treatment appears to reflect a multidimensional survivorship outcome influenced by interacting psychosocial, behavioral, clinical, and treatment-related factors. Integrating these dimensions into postoperative risk assessment may support earlier identification of patients at increased risk and promote more personalized rehabilitation-oriented survivorship care.

## Informed consent statement

Informed consent was obtained from all subjects involved in the study. The prospective implementation cohort was registered at ClinicalTrials.gov (NCT05950685) prior to participant enrollment.

## Ethics approval and consent to participate

The study was conducted in accordance with the Declaration of Helsinki and approved by the Institutional Review Boards of Assuta Medical Centers (protocol codes ASMC-0019-22, ASMC-0018-23).

## Data availability statement

The datasets generated and analyzed during the current study contain potentially identifiable patient information and are therefore not publicly available. De-identified data may be available from the corresponding author upon reasonable request and subject to institutional review board approval and applicable privacy regulations.

## Declaration of generative AI and AI-assisted technologies in the manuscript preparation process

During the preparation of this work, the authors used ChatGPT (OpenAI) to assist with language editing, text refinement, organizational structuring of the manuscript, and support for statistical coding workflows. After using this tool, the authors critically reviewed, revised, and edited all content as needed and take full responsibility for the content of the published article.

## Funding

This research was supported by the Israel Cancer Association (Grant No. 20240007) and Office of the Chief Scientist, Ministry of Health, Israel (Grant No. 3-18513).

## CRediT authorship contribution statement

**Ifat Klein:** Conceptualization, Data curation, Formal analysis, Resources, Validation, Visualization, Writing – original draft, Writing – review & editing. **Danit R. Shahar:** Conceptualization, Methodology, Validation, Visualization, Writing – original draft, Writing – review & editing. **Michael Friger:** Conceptualization, Formal analysis, Methodology, Supervision, Validation, Visualization, Writing – original draft, Writing – review & editing. **Irena Rosenberg:** Data curation, Methodology, Resources, Writing – original draft, Writing – review & editing. **Sergio Susmallian:** Conceptualization, Investigation, Methodology, Validation, Visualization, Writing – original draft, Writing – review & editing. **Daphna Barsuk:** Data curation, Investigation, Writing – original draft, Writing – review & editing. **Merav A. Ben-David:** Conceptualization, Data curation, Funding acquisition, Investigation, Methodology, Resources, Supervision, Validation, Visualization, Writing – original draft, Writing – review & editing.

## Declaration of competing interests

The authors declare that they have no known competing financial interests or personal relationships that could have appeared to influence the work reported in this paper.
