# Supplementary material for: Beyond surgical risk: Multidimensional predictors of postoperative arm morbidity after breast cancer treatment
Source: Breast. 2026 Jun 20;88:104846. doi: 10.1016/j.breast.2026.104846 (PMC13316302; doi:10.1016/j.breast.2026.104846)
Supplement: Multimedia component 1 [file mmc1.docx]

**SUPPLEMENTARY DATA**

**Supplementary Figure S1.** Study flowchart of participant selection and inclusion


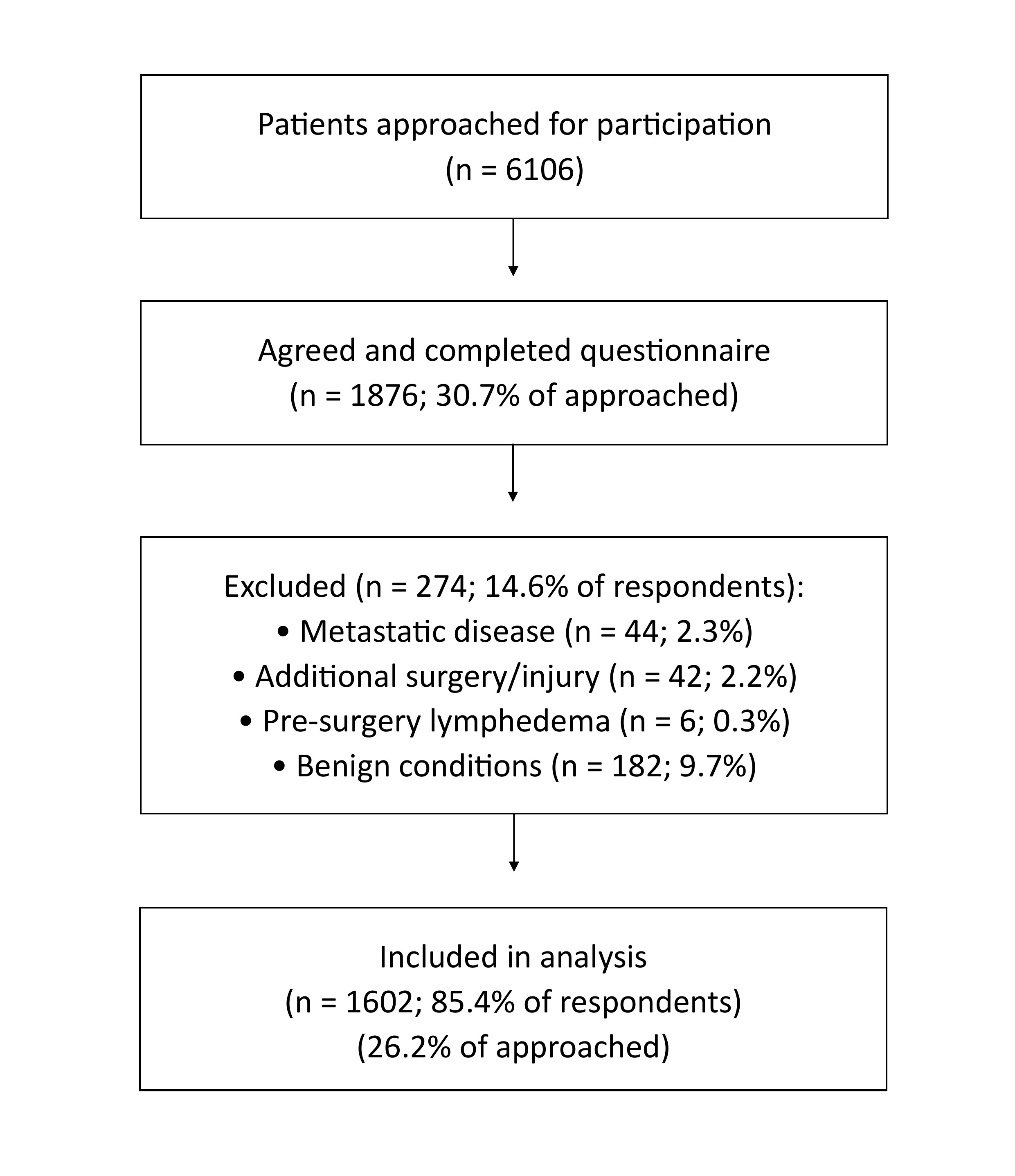


Flowchart illustrating the selection and inclusion of participants in the pooled cohort. The figure outlines eligibility criteria, exclusions, and the final analytic sample.

**Supplementary Table S1.** Feature importance of predictors in the XGBoost model

| **Variable** | **Importance** |
| --- | --- |
| Insomnia | 0.285 |
| Physical activity level | 0.098 |
| Hormonal therapy | 0.092 |
| Disease stage | 0.080 |
| Comorbidity | 0.073 |
| Chemotherapy | 0.060 |
| Physiotherapy during hospitalization | 0.050 |
| Psychosocial distress | 0.044 |
| Radiation therapy | 0.042 |
| Postoperative complications | 0.033 |
| Surgical type (mastectomy vs. lumpectomy) | 0.029 |
| Biological therapy | 0.023 |
| Age | 0.021 |
| Body mass index (BMI) | 0.019 |
| Severe pain during hospitalization | 0.015 |
| Extent of lymph node dissection | 0.014 |
| Breast reconstruction | 0.013 |
| Lack of family support | 0.011 |

*Feature importance values represent the relative contribution of each predictor to the model’s classification performance, based on their role in improving split quality across decision trees. These values are relative and do not indicate direction of effect. Importance values do not reflect the direction of association but rather the magnitude of contribution to model prediction.*
